# Comparative Study of Experimental and Theoretical Evaluation of Nocturnal Cooling System for Room Cooling for Clear and Cloudy Sky Climate

**DOI:** 10.1002/gch2.201900008

**Published:** 2019-07-11

**Authors:** Pramod V. Mulik, Uday C. Kapale, Gautam S. Kamble

**Affiliations:** ^1^ Department of Mechanical Engineering Tatyasaheb Kore Institute of Engineering & Technology (TKIET) Warananagar Warananagar 416113 Maharashtra India; ^2^ Department of Mechanical Engineering SSET's S.G Balekundri Institute of Technology (SGBIT) Belgaum Belgaum 590010 Karnataka India

**Keywords:** active cooling system, nocturnal cooling radiator, passive cooling, radiative cooling

## Abstract

Nocturnal radiation is one of the effective passive cooling technologies by infrared radiation exchange between earthly surfaces and the sky. Heating ventilation and air conditioning (HVAC) systems are highly energy‐demanding and there is a need to develop alternative low‐energy means to achieve comfort. Radiative cooling reduces the electricity requirements, which normally is generated through fossil fuels, in order to run active cooling systems. During the day, this is offset by solar radiation gains on the roof; however, at night, this heat loss has the ability to cool air, as roofs can experience a temperature drop of 2–12 °C below ambient. The experimental investigation of the aluminum material and their surface colors on nocturnal cooling is presented for clear and cloudy sky conditions. The material of aluminum and black color coating are considered. Results obtained reveal that the plates' performances greatly depend on the presence of clouds in the night. The best results are obtained in a clear sky summer climate condition than in the cloudy sky condition. The net cooling rate of night sky radiation system for without and with coating on aluminum of mass flow rate 0.05 kg s^−1^ is near about 72.30–80.99 W m^−2^ for clear sky and 48.75–53.25 W m^−2^ for cloudy sky condition.

## 1 Introduction

Worldwide energy reduction is rapidly increasing and is likely to rise all over again.^1^ The decline of conventional sources, which is near about 80% of basic energy sources,^2^ and the ecological blow related with its uses have attracted us toward our interest to energy protection. The overall energy utilization for building thermal comfort accounts for 40%. For particular cooling systems, provides a huge possibility for energy conservation.^3^

In hot and humid climates, cooling is a necessary part of human in conventional cooling system of the majority buildings. In reality, due to huge usage of energy in buildings, it has increased burden to propose HVAC equipments and systems energy economically. These cooling systems consume great quantity of electrical power.^2^ Due to global warming, the issues of climatic change occur. The replacement of conventional active cooling system consumes large amount of energy by the use of eco‐friendly resources. The usage of comfort cooling the use of night cooling system is considered. Also, this system is identified due to its zero pollution, uncomplicated maintenance, small energy use, simplicity, and good quality interior air.

## 2 Literature Review

A lot of work has been done on the night cooling phenomenon. The purpose of this literature review is to go through the core topics of awareness. The make another study of literature is concerned with passive cooling and different radiator materials with theoretical and experimental evaluation. Lombard et al.^1^ have discussed about the fast rising globe energy use that has by now raised concerns over supply problems, exhaustion of energy resources, and deep ecological impacts. The energy consumption for residential and commercial is near about 20% and 40% in urban countries. Increase in the requirement for comfort level in building services rise due to increase in population, so that the demand for trends in energy continues in coming generation. Kolokotsa et al.^3^ have focused on buildings that are gradually expected more to meet advanced compound performance needs. Their study reveals that it is sustainable, use no energy, provide a comfortable atmosphere for the occupants, cost‐effective to construct and sustain. The necessary factors for the unbeaten growth and operation of net zero and constructive energy buildings are recreation models that are correct presentations of the construction of buildings. Farmahini‐Farahani and Heidarinejad^4^ have examined the possibility of this system to four cities that have different surrounding situation. The water from a storage tank has circulated through two radiative panels for whole night in clear sky condition. Ghassem Heidarinejad et. al. have developed a hybrid system and complements direct evaporative cooling as if it consumes low energy to provide cold water and is able to fulfill the comfort condition, whereas direct evaporative alone is not able to provide summer comfort condition.^5^ Muselli^6^ has elaborated that during hot season in diurnal time the heat gains are allowed by inexpensive new materials. To assess the importance of these new materials of UV–vis–IR performance is considered and compared to usual roof resources existing in industrial and growing countries. The surface temperature 60 °C of uncoated material simulations showed that the economical white opaque roofs (50 m^2^) presented in this research should decrease cooling energy utilization by 26–49%. Parker and Sherwin^7^ focused on a nocturnal cooling system developed, significantly there is decrease in room comfort requirements in North American environment. The model uses a sealed attic enclosed by a well‐conductive metal roof which is selectively related by air flow to the main region with the attic region to provide cooling mostly during night time. Parker and Sherwin^8^ have performed experimental assessment on a night sky cooling system. It was observed that performance was lower than the earlier simulation analysis. Authors found that every day run period fraction throughout which the night cool exhaust fan operated between from 12% to 36% in August–September and May, respectively. The inference was that over 6 h functional time, which should generate about 0.2 ton h of sensible cooling. Bagiorgas and Mihalakakou^9^ have focused that the aluminum radiator is used for cooling the ambient air below its initial temperature. By using perfect mathematical model, the active thermal performance of the system during summer climate is calculated, based on the heat transferred to the ambient air from the air inside the radiator. Hollick in Nocturnal radiation cooling tests^10^ has summarized that the sun and earth radiate heat to earth and sky in the day and night, respectively. The solar radiation gains by the roofs at day time, and on the other hand, at night time, earth radiates heat to the sky, and due to this heat loss has the facility to cool air as roofs can occur at a temperature decrease from 2 to 12 °C below surrounding temperature.

In this paper, experimental and theoretical models are presented to predict the potential of radiative cooling for clear sky and cloudy sky climate of western Maharashtra. The cooling requirements for the western Indian climatic conditions are from the month of March to June. The months from July to February are monsoon and winter which do not require cooling in these months. So, in this paper, we have presented the comparative study of the radiative cooling potential in the clear sky condition in summer days and cloudy sky rainy climate condition.

## 3 Design of Nocturnal Cooling System

**Figure 1:**
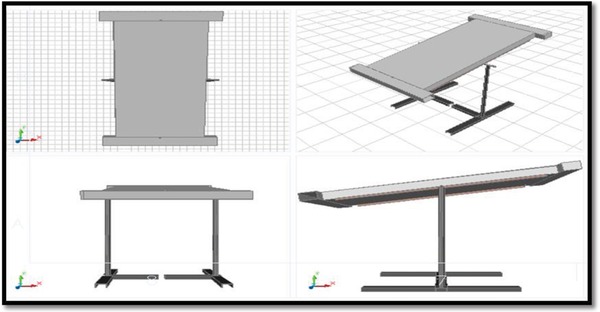
Heat exchange unit with mounting stand.

By studying the literature survey, we understand that there are difficulties that arrived during the installation of the nocturnal concept to any building; because it requires some prerequisites like metal roofing. To overcome this problem, we prepared a system like flat plate collector which can be easily installed to any building. It works as follows: The air inside the room is sucked and tries to flow forcefully through the duct by the one exhaust fan. The duct is internally connected to the inlet of heat exchanger. The control valve is used to control the air mass flow rate in between the duct and heat exchanger unit (**Figure** **1**).

The heat exchanger has two aluminum sheet plates of 0.44 mm thickness separated by a gap of 10 cm. When the air flows through this gap, the heat from flowing air is removed by the plates with increase in temperature; but the flowing air loses the heat with decrease in temperature. This cooled air is again fed back to the room. The heated plate loses the heat to the surrounding atmosphere due to the temperature gradient between them. By circulating the air again and again, the room is brought to the comfort temperature. In this way, the nocturnal cooling system uses the naturally obtained temperature gradient as renewable energy source.

Various papers on night cooling systems are studied, by reference of which cad model is formed and the actual model is also built. Some considerations are taken into account while constructing the model. Required data for the construction is collected from papers and some references. Fabrication work was completed by preparing the separate subparts and assembling them. Those subparts are as follows.

**Figure 2:**
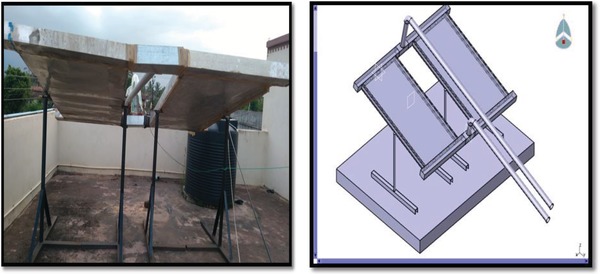
Actual nocturnal cooling system.

1) Heat exchanger unit. 2) Mounting stand. 3) Duct. 4) Piping connections. 5) Exhaust fan. 6) Temperature measurement by using probe type thermocouple. 7) Velocity measurement by anemometer (**Figure** **2**).

## 4 Theoretical Analysis of System

By using correct mathematical equations, the energetic thermal performance of the heat exchanger has been calculated. Total heat removed from radiator circulating air by the system is total radiant energy

(1) $$Q_{\mathrm{total}}\;=\;Q_{\mathrm{top}}+Q_{\mathrm{bottom}}$$

The change in internal energy of the circulating air in radiator is useful heat and the air flow is calculated

(2) $$Q_{\mathrm{useful}}\;=\;mcp\left(T_{\mathrm{in}}-\;\;T_{\mathrm{out}}\right)$$

Efficiency of the radiator system is given by in Equation 3.

The effectiveness of the heat exchanger unit is the ratio of the useful change in internal energy to the total energy output from the surfaces of heat exchanger unit

(3) $$n_{\mathrm{radiator}}\;=\;\frac{Q_{\mathrm{useful}}}{Q_{\mathrm{total}}}$$

Heat loss from top surface of radiator to sky and surrounding is given by

(4) $$Q_{\mathrm{top}}\;=\;U_{\mathrm{top}}A_{\mathrm{top}}\left(T_{\mathrm{fin}}-\;\;T_{\mathrm{atm}}\right)$$

Similarly, heat loss from bottom surface of radiator to surrounding is

(5) $$Q_{\mathrm{bottom}}\;=\;U_{\mathrm{bottom}}A_{\mathrm{bottom}}(T_{\mathrm{fin}}-\;\;T_{\mathrm{atm}})$$

Here, overall heat transfer coefficient from top and bottom surface of radiator is calculated by the following Equations (4) and (5)

(6) $$U_{\mathrm{top}}\;=\;1/R_{\mathrm{total}\;\mathrm{top}}$$

(7) $$U_{\mathrm{bottom}}\;=\;1/R_{\mathrm{total}\;\mathrm{bottom}}$$

For above Equations (4) and (5), we are required to calculate total resistance offered for heat transfer from top surface and bottom surface

(8) $$R_{\mathrm{total}\;\mathrm{top}}\;=\;R_{\mathrm{top}}+R_{\mathrm{conduction}\;\mathrm{top}}+R_{\mathrm{convection}\;\mathrm{air}}$$

(9) $$R_{\mathrm{total}\;\mathrm{bottom}}\;=\;R_{\mathrm{conv}\;\mathrm{wind}\;\mathrm{bottom}}+R_{\mathrm{cond}\;\mathrm{bottom}}+R_{\mathrm{conv}\;\mathrm{air}}$$

### 4.1 Heat Loss from Top Surface of the Radiator

#### 4.1.1 Convective Heat Loss from Surface to Surrounding Air

Convective heat loss from surface to surrounding air is calculated by^11^

(10) $$h_{\mathrm{convection}\;\mathrm{top}}\;=\;2.8+4.8\;V_{\mathrm{wind}}$$

where *V*_wind_ is the velocity of surrounding air in m s^−1^

#### 4.1.2 Heat Loss by Radiation from Top Surface to Sky

Heat loss by radiation from top surface to sky is calculated by

(11) $$h_{\mathrm{rad}\;\mathrm{sky}}\;=\;\sigma *\varepsilon *\left(T_{r}-T_{\mathrm{sky}}\right)*\left(T_{r}+T_{\mathrm{sky}}\right)*\left(T_{r}{}^{2}+T_{\mathrm{sky}}^{2}\right)/\left(\left(T_{r}-T_{\mathrm{sky}}\right)\right)$$

where σ is Stefan Boltzman's constant, ε is emissivity of the surface, *T*_r_ is radiator surface average temperature in K, and *T*_sky_ is sky temperature in K.

#### 4.1.3 Sky Temperature Calculations

Effect of cloudiness: CC is 0, for clear climate; 1, for fully cloudy climate.

Air temperature *T* air in K

Dew point temperature *T*_dp_ in °C

Model for clear sky

(12) $$T_{\mathrm{clear}\;\mathrm{sky}}\;=\;T_{\mathrm{air}}{\left(\varepsilon_{\mathrm{clear}}\right)}^{0.25}$$

where, ε_clear_, emissivity of clear sky is calculated by the following equation

(13) $$\varepsilon_{\mathrm{clear}}\;=\;0.711+0.56\left(T_{\mathrm{dp}}/100\right)+0.73{\left(T_{\mathrm{dp}}/100\right)}^{2}$$

$$\mathrm{Ca}\;=\;1+0.0224\mathrm{CC}+{\left(0.0035\mathrm{CC}\right)}^{2}+{\left(0.00028\mathrm{CC}\right)}^{3}+.........$$

where CC is effect of cloudiness.

Now, for calculating temperature of sky by using the following equation

(14) $$T_{\mathrm{sky}}\;=\;{\left(\mathrm{Ca}\right)}^{0.25}\times \;\;\;T_{\mathrm{clear}\;\mathrm{sky}}$$

The temperature of sky in cloudy condition is calculated by the following expression,

Model for cloud sky

(15) $$T_{\mathrm{sky}}^{4}\;=\;9.365574*10^{-6}\left(1-\mathrm{CC}\right)Ta^{6}+Ta^{4}\mathrm{CC}*\varepsilon_{\mathrm{cloudiness}}$$

The emissivity of cloudy sky is calculated as

(16) $$\varepsilon_{\mathrm{cloudiness}}\;=\;\left(1-0.84\mathrm{CC}\right)\left(0.527+0.161\;e^{\left[8.45\left(1-\frac{273}{T_{\mathrm{air}}}\right)\right]}+0.84\mathrm{CC}\right)$$

#### 4.1.4 Thermal Resistance Offered for Radiation Mode of Heat Transfer and Convective Mode of Heat Transfer

Thermal resistance offered for radiation mode of heat transfer and convective mode of heat transfer is given by

(17) $$R_{\mathrm{top}}\;=\;\frac{1}{h_{\mathrm{rad}\;\mathrm{sky}}\;\;+\;\;\;h_{\mathrm{convective}}}$$

#### 4.1.5 Thermal Resistance Offered by Plate to Conduction Mode of Heat Transfer

Thermal resistance offered by plate to conduction mode of heat transfer is calculated by

(18) $$R_{\mathrm{conduction}\;\mathrm{top}}\;=\;\frac{tp}{Kp}$$

#### 4.1.6 Thermal Resistance Offered for Convective Heat Transfer between Circulating Air and Plate

Thermal resistance offered for convective heat transfer between circulating air and plate is given by

(19) $$R_{\mathrm{convective}\;\mathrm{air}}\;=\;\frac{1}{h_{\mathrm{air}}}$$

### 4.2 Heat Loss from Bottom Surface of the Radiator

#### 4.2.1 Convective Heat Loss from Bottom to Surrounding

Convective heat loss from bottom to surrounding is considered by

(20) $$R_{\mathrm{convection}\;\mathrm{wind}\;\mathrm{bottom}}\;=\;\frac{1}{h_{\mathrm{convection}\;\;\mathrm{bottom}}}$$

where

(21) $$h_{\mathrm{convection}\;\mathrm{bottom}}\;=\;2.8+4.8\;V_{\mathrm{wind}}$$

#### 4.2.2 Heat Transfer through the Plate of Radiator

Heat transfer through the plate of radiator is considered by the equation given below

(22) $$R_{\mathrm{conduction}\_\mathrm{bottom}}\;=\;\frac{tp}{kp}$$

#### 4.2.3 Heat Transfer by Air to the Bottom Structure and Edge due to Convection with Duct

Heat transfer by air to the bottom structure and edge due to convection with duct is given by

(23) $$R_{\mathrm{conductive}\;\mathrm{bottom}}\;=\;\frac{1}{h_{\mathrm{air}}}$$

## 5 Results and Discussion

The research of nocturnal cooling has been accomplished in the months of May, August, and September, i.e., in clear and cloudy sky season. We performed our experimental work in two climate conditions as well as two different parameters like change in the emissivity of radiator by black coating and variation in mass flow rate provided to radiator. For performing this experimental work, we had taken 40 days observations for two different conditions. A heat exchanger unit with the total surface area of 4 m^2^ was examined. Internal cooling load is dependent on human being and electrical devices, and is independent from the climate. The data contain parameters such as each day ambient temperature, relative humidity, and wind speed. In order to calculate the radiative cooling potential, the important parameters are ambient temperature and relative humidity. Using these parameters, sky temperature is calculated which will be used to calculate the cooling power.

**Figure 3:**
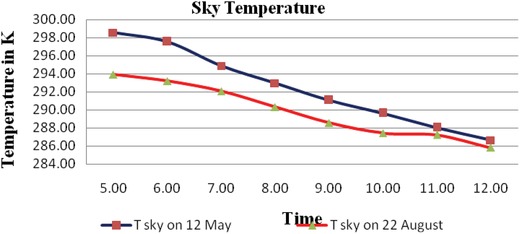
Time versus sky temperature in clear and cloudy conditions.

After measuring different parameters as mentioned in **Tables** **1** and **2**, the study is to evaluate the radiative cooling potential. The sky emissivity is calculated using Equation (13). It is found that the emissivity of sky varies from 0.896 to 0.856 in the month of May and 0.891 to 0.852 in the month of August. The sky temperature and ambient temperature difference is 8–15 °C in the month of May and August and attains a maximum value of 15.8 °C in the rainy month of June and July. The **Figure** **3** shows time versus sky temperature in clear and cloudy day.

**Table 1:** Measuring parameters such as temperature, wind velocity, and humidity on 12 May

Sr. No.	Time	Temperature of radiator [K]	Temperature of air [K]	Velocity of wind [m s^−1^]	Humidity (RH)	Cloudiness(CC)
1	5	306.80	306.80	3.576	56.00	0.00
2	6	305.50	305.70	3.576	62.00	0.00
3	7	302.80	303.90	4.470	65.00	0.00
4	8	301.50	302.60	4.470	67.00	0.00
5	9	300.40	301.50	4.470	67.00	0.00
6	10	299.20	300.40	4.023	70.00	0.00
7	11	298.10	299.20	4.023	73.00	0.00
8	12	296.70	298.00	3.576	76.00	0.10

**Table 2:** Measuring parameters such as temperature, wind velocity, and humidity on 22 August

Sr. No.	Time	Temperature of radiator [K]	Temperature of air [K]	Velocity of wind [m s^−1^]	Humidity (RH)	Cloudiness (CC)
1	5	301.10	301.40	6.705	80.00	0.60
2	6	299.20	300.90	6.258	81.00	0.59
3	7	298.90	299.90	5.364	85.00	0.60
4	8	298.00	298.90	5.364	85.00	0.58
5	9	297.40	297.70	4.470	87.00	0.57
6	10	296.60	296.90	4.023	88.00	0.62
7	11	295.80	296.70	4.023	88.00	0.69
8	12	294.80	295.80	4.023	88.00	0.75

**Figure 4:**
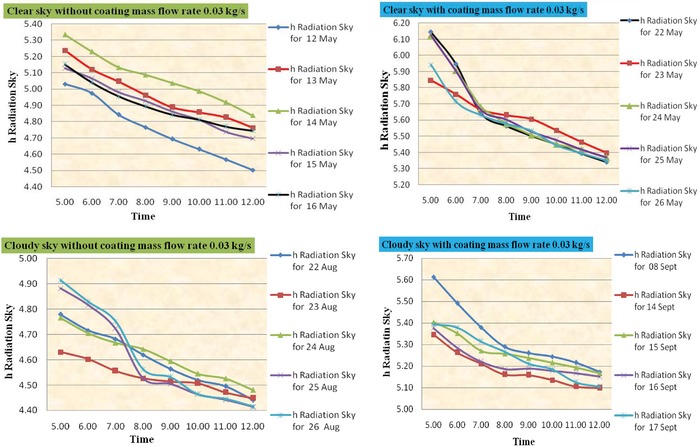
Time versus *h*_radiation_ (without and with coating for mass flow rate 0.03 kg s^−1^).

The radiator's radiation heat loss coefficient is dependent on ambient temperature and emissivity of the radiator; according to Equation (11), is an important factor for dissipating the excess heat from the top surface. In the cooling application, the radiator's emissivity is expected to be between 0.80 and 0.94. **Figure** **4** illustrates the slight effect of this parameter on the radiative coefficient in different ambient temperatures, as determined through theoretical calculations. It has been observed that the radiative heat transfer coefficient can be varied in the range 6.19–4.54 W m^2^ K^−1^ for clear sky condition and in the range 5.60–4.40 W m^−2^ K^−1^) for cloudy sky condition.

**Figure 5:**
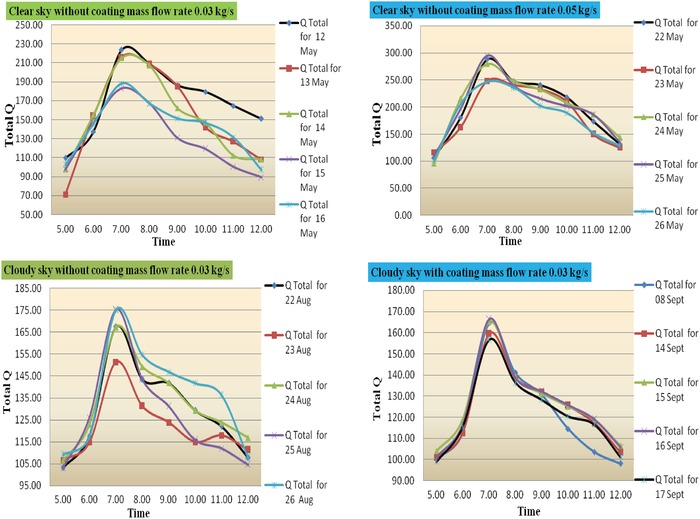
Time versus net cooling power (without and with coating for mass flow rate 0.03 kg s^−1^).

The net cooling rates are the functions of the properties of the radiator material, on the environmental conditions, on the fluid inlet temperature, and mass flow rate. Also, it has been observed that in **Figure** **5** the values of net cooling range for without coating and coating on aluminum radiator is from 55.84 to 71.88 and 72.30 to 88.99 W m^−2^ as the mass of air flow increases from 0.03 to 0.05 kg s^−1^, respectively, for clear sky condition. Also it has been observed that the values of net cooling range for without coating and coating on aluminum radiator is from 41.25 to 53.25 and 38.75 to 48.75 W m^−2^ as the mass of air flow increases from 0.03 to 0.05 kg s^−1^, respectively.

**Figure 6:**
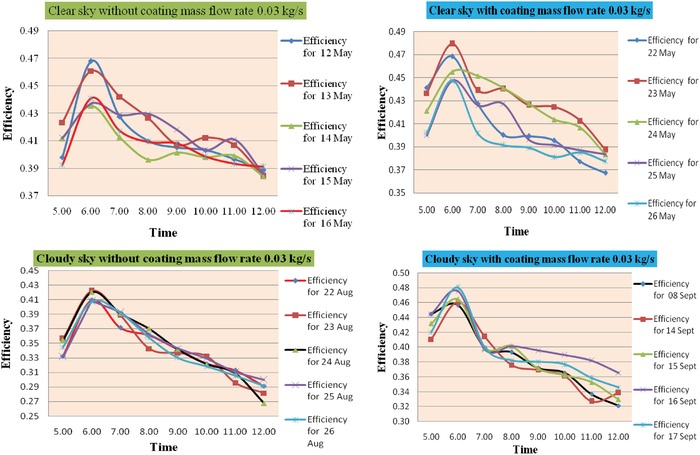
Time versus efficiency (without and with coating for mass flow rate 0.03 kg s^−1^).

The efficiency of heat exchanger unit is determined by measuring parameters by using Equation (3). It is observed that in **Figure** **6** the values of efficiency range for without coating on aluminum radiator is from 0.41 to 0.50 as the mass flow rate is 0.05 kg s^−1^ and 0.34 to 0.47 as the mass flow rate is 0.03 kg s^−1^. And values of efficiency range for without coating on aluminum radiator is from 0.36 to 0.47 as the mass flow rate is 0.05 kg s^−1^ and 0.26 to 0.41 as the mass flow rate is 0.03 kg s^−1^.

## 6 Conclusion

A review of nocturnal cooling system has been undertaken. From this comparative study, the following are evident:

### 6.1 For Clear Sky Climate Condition

1) The net cooling rate of night sky radiation system for with and without coating on aluminum of mass flow rate 0.05 kg s^−1^ is near about 72.30–80.99 W m^−2^.
2) The net cooling rate of night sky radiation system for with and without coating on aluminum of mass flow rate 0.03 kg s^−1^ is near about 55.84–71.88 W m^−2^.
3) Efficiency of heat exchanger unit varies from 0.41 to 0.50 and 0.34 to 0.47 as the mass of air flow is 0.05 and 0.03 kg s^−1^, respectively.

### 6.2 For Cloudy Sky Climate Condition

1) The net cooling rate of night sky radiation system for with and without coating on aluminum of mass flow rate 0.05 kg s^−1^ is near about 48.75–53.25 W m^−2^.
2) The net cooling rate of night sky radiation system for with and without coating on aluminum of mass flow rate 0.03 kg s^−1^ is near about 38.75–41.25 W m^−2^.
3) Efficiency of heat exchanger unit varies from 0.36 to 0.47 and 0.26 to 0.41 as the mass of air flow is 0.05 and 0.03 kg s^−1^, respectively.
4) With the nocturnal cooling system, the cooling energy demand of a building can be reduced as much as 28%.
5) There is a need for increased awareness on space cooling by nocturnal radiation since it has the potential of greatly reducing green‐house gas emissions.

## Conflict of Interest

The authors declare no conflict of interest.
